# Congenital Malalignment of the Great Toenails: A Rare Familial Presentation in Adolescence

**DOI:** 10.7759/cureus.96556

**Published:** 2025-11-11

**Authors:** Abdulrahman Saleh Aldairi, Faris Alsaedi, Homaid Alotaibi

**Affiliations:** 1 Department of Dermatology, King Faisal Hospital, Ministry of Health, Makkah, SAU

**Keywords:** congenital malalignment, familial inheritance, great toenail, nail dystrophy, onychocryptosis, onychomadesis, onychomycosis mimic, toenails

## Abstract

Congenital malalignment of the great toenail (CMGT) is a rare nail disorder characterized by lateral deviation of the nail plate due to malrotation of the nail matrix. We describe the case of a female adolescent with progressive nail discoloration, abnormal growth, and recurrent shedding of the great toenails. Similar findings were also observed in other family members, suggesting a hereditary predisposition. Fungal studies were negative. The patient was managed conservatively with protective footwear and regular trimming, while surgical matricectomy was declined. This case underscores the importance of distinguishing CMGT from onychomycosis and highlights the value of conservative management in the absence of severe symptoms.

## Introduction

Congenital malalignment of the great toenail (CMGT) is a rare nail disorder characterized by lateral or, less frequently, medial deviation of the nail plate due to malrotation of the nail matrix relative to the distal phalanx [1]. The condition often presents at birth or during early childhood, though in some cases, it becomes more apparent later in life due to mechanical stress [2]. Its prevalence is estimated at 1-2% among children, though the true rate is likely underreported [3]. CMGT may occur sporadically or show familial clustering, suggesting an autosomal dominant inheritance with variable expressivity [4]. Clinically, it can mimic onychomycosis, with features such as discoloration, thickening, and nail dystrophy leading to delayed or incorrect diagnosis [5]. While mild cases may improve spontaneously, persistent or severe disease can result in complications, including onychocryptosis, paronychia, retronychia, or onychomadesis [6]. This report describes a familial case of CMGT with recurrent nail shedding in an adolescent female, emphasizing diagnostic pitfalls and management strategies.

## Case presentation

A 15-year-old otherwise healthy female presented to the dermatology clinic with her mother, complaining of yellow discoloration and abnormal growth of both great toenails, a condition that had been present since early childhood. She had previously attended several private clinics, where she was prescribed oral terbinafine and amorolfine 5% nail lacquer for presumed onychomycosis, but without improvement. There was no history of trauma, joint pain, systemic illness, or other dermatological conditions. She was not on any long-term medications.

Family history was notable, as her father and younger sister (aged 12 years) reportedly experienced similar nail abnormalities, suggesting a hereditary predisposition. However, direct examination and photography of these family members were not possible, as the patient lived with her mother while her sister resided with her father. The mother showed us personal photographs of her husband and younger daughter exhibiting similar nail changes, but consent for publication was obtained only from our patient and her mother.

**Figure 1:**
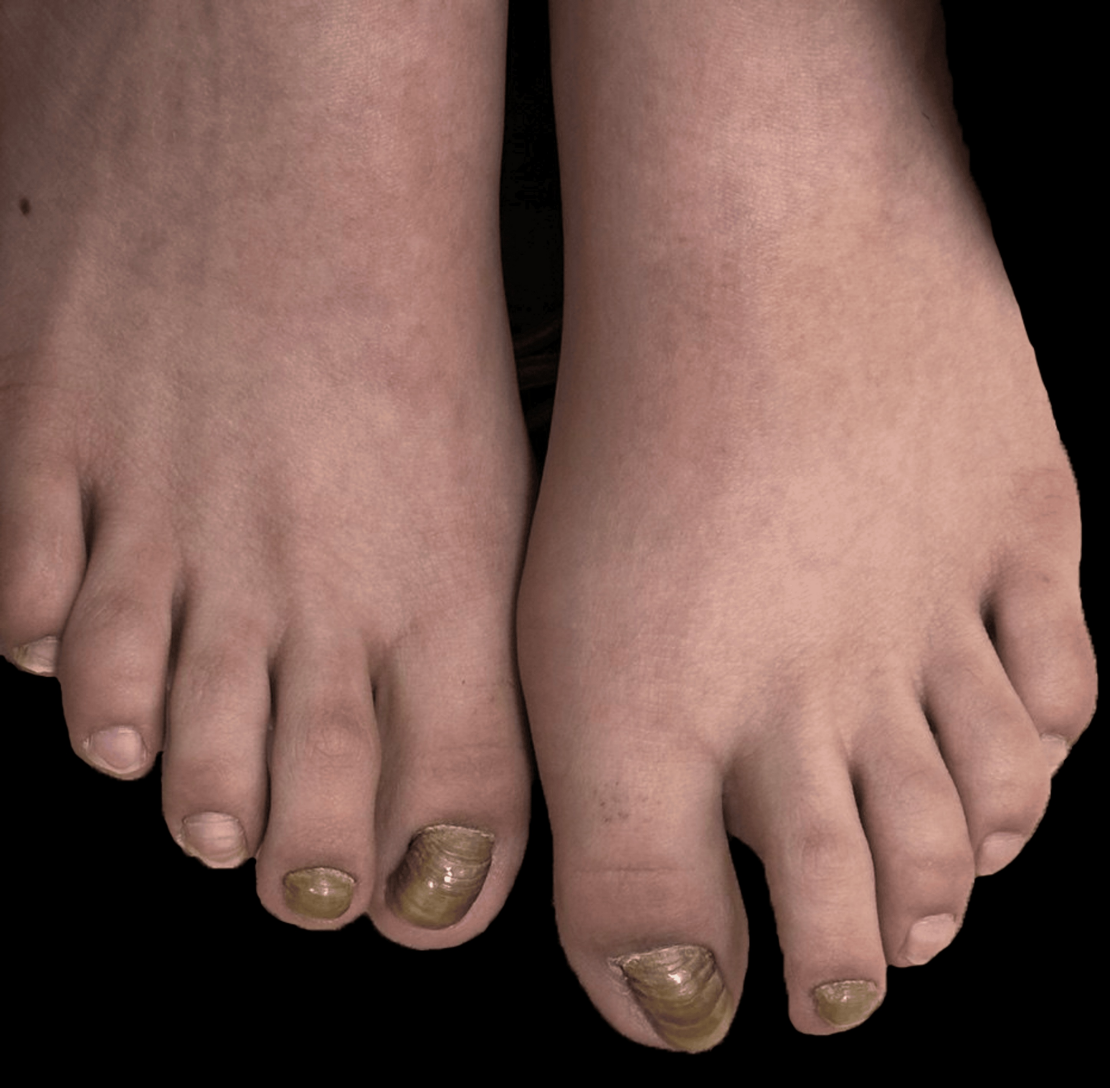
Big and second toenails of both feet showing nail thickening with multiple transverse grooves and ridges, along with yellow-brown discoloration. The great toenails additionally demonstrate increased curvature and lateral deviation of the nail plates.

On examination, both great toenails displayed marked yellow discoloration, increased transverse curvature, and lateral deviation of the nail plates relative to the distal phalanx, consistent with CMGT. Yellowish discoloration was also present on both second toenails, though without significant thickening or deviation (Figure 1).

According to the mother and patient, the affected nails underwent repeated cycles of abnormal growth followed by spontaneous shedding and regrowth. There was no periungual erythema, swelling, or tenderness, and the other toenails appeared normal. Potassium hydroxide (KOH) preparation and fungal culture from nail clippings were negative for fungal elements, thereby excluding onychomycosis. A nail biopsy was advised for further confirmation, but the patient and her mother declined. She was counseled regarding conservative management, including protective footwear and regular trimming. Surgical intervention with total nail avulsion and matricectomy was discussed but not pursued, as her mother preferred conservative care. At a six-month follow-up, the patient remained asymptomatic, and the nail deformity was stable without evidence of pain, inflammation, or infection.

## Discussion

CMGT remains an underrecognized but clinically relevant cause of chronic nail dystrophy in pediatric and adolescent populations [1]. Our patient exhibited classical signs of yellow discoloration, curvature, and lateral deviation, consistent with prior reports [2]. The presence of similar nail findings in her father and sister suggests hereditary involvement, aligning with earlier studies that documented familial clustering and variable penetrance [4]. The cyclical shedding of the patient’s great and second toenails corresponds to onychomadesis, which has been described as a complication of CMGT [6]. Similar cases of spontaneous nail shedding and regrowth without fungal infection have been reported, highlighting the role of microtrauma and abnormal biomechanics in disrupting nail matrix function [6]. Misdiagnosis as onychomycosis is common, and our patient’s history of multiple antifungal treatments without improvement reflects this diagnostic challenge. In such cases, negative fungal studies and therapeutic failure are key diagnostic clues [7]. Management of CMGT varies depending on severity and patient preference. Conservative measures, such as protective footwear, proper trimming, and trauma avoidance, are recommended for mild or asymptomatic disease [1]. Surgical options, including nail avulsion with matricectomy, may be reserved for severe deformity or functionally limiting complications [2]. In the present case, the patient and family preferred reassurance and conservative care, which is consistent with recommendations for stable disease [8].

## Conclusions

CMGT is a rare but significant cause of nail dystrophy that can easily be mistaken for onychomycosis. Recognizing its characteristic features and familial tendency is essential to avoid unnecessary treatments. Most cases can be managed conservatively, while surgery is reserved for severe or symptomatic presentations. Early diagnosis ensures appropriate care and better patient outcomes.
